# *Helicobacter pylori* infection alters gastric and tongue coating microbial communities

**DOI:** 10.1111/hel.12567

**Published:** 2019-02-07

**Authors:** Yubin Zhao, Xuefeng Gao, Jiaxuan Guo, Dongbao Yu, Ying Xiao, Huijie Wang, Yuchan Li

**Affiliations:** ^1^ The Traditional Chinese Medicine Hospital of Shijiazhuang Affiliated to Hebei University of Chinese Medicine Shijiazhuang China; ^2^ Shenzhen University General Hospital Shenzhen China; ^3^ Shenzhen University Clinical Medical Academy Shenzhen China; ^4^ Shenzhen Hoiracle Bio‐Tech Co., Ltd. Shenzhen China; ^5^ Department of Bioengineering, School of Chemical Engineering Shijiazhuang University Shijiazhuang China

## Abstract

**Objective:**

Infection with *Helicobacter pylori* (*H pylori*), especially cytotoxin‐associated gene A‐positive (CagA+) strains, has been associated with various gastrointestinal and extragastric diseases. The aim of this study was to characterize *H pylori*‐induced alterations in the gastric and tongue coating microbiota and evaluate their potential impacts on human health.

**Design:**

The gastric mucosa and tongue coating specimens were collected from 80 patients with chronic gastritis, and microbiota profiles were generated by 16S rRNA gene sequencing. Samples were grouped as *H pylori negative* (n = 32), CagA‐negative *H pylori infection* (n = 13), and CagA‐positive *H pylori infection* (n=35). The comparison of bacterial relative abundance was made using a generalized linear model. Functional profiling of microbial communities was predicted with PICRUSt and BugBase. Microbial correlation networks were produced by utilizing SparCC method.

**Results:**

Significant alterations of the gastric microbiota were found in the *H pylori*+/CagA+ samples, represented by a decrease in bacterial diversity, a reduced abundance of *Roseburia*, and increased abundances of *Helicobacter* and *Haemophilus* genera. At the community level, functions involved in biofilm forming, mobile element content, and facultative anaerobiosis were significantly decreased in gastric microbiome of the *H pylori*+ subjects. The presence of CagA gene was linked to an increased proportion of Gram‐negative bacteria in the stomach, thereby contributing to an upregulation of lipopolysaccharide (LPS) biosynthesis. The number of bacterial interactions was greatly reduced in networks of both tongue coating and gastric microbiota of the *H pylori*+/CagA+ subject, and the cooperative bacterial interactions dominated the tongue coating microbiome.

**Conclusions:**

Infection with *H pylori* strains possessing CagA may increase the risk of various diseases, by upregulating LPS biosynthesis in the stomach and weakening the defense of oral microbiota against microorganisms with pathogenic potential.

## 1 INTRODUCTION

Highly acidic condition together with various antimicrobial chemicals and enzymes make stomach a hostile environment for most microorganisms. However, investigations of gastric fluid and biopsy samples by next‐generation sequencing (NGS) demonstrate that the human gastric microbiota is a diverse ecosystem that dominated by five phyla, including Actinobacteria, Bacteroidetes, Firmicutes, Fusobacteria, and Proteobacteria.^1^, ^2^ There is a trend of gastric microbiota alterations in people with *Helicobacter pylori* (*H pylori*) infection; however, inconsistent results have been obtained.^3^, ^4^, ^5^, ^6^ With a small Colombian patient cohort (n = 40), Yang et al showed that the overall gastric microbiota composition was largely independent of the *H pylori* infection and carriage of the cag pathogenicity island.^3^ In contrast, another study with a small cohort (n = 30) demonstrated that the abundances of several genera in the gastric microbial community are significantly different between *H pylori* negative and *H pylori *positive individuals.^4^ Infection with *H pylori *is a leading risk factor for the development of gastric cancer*,* making it a type I carcinogen.^7^ However, only approximately 3% of *H pylori*‐infected patients developed gastric cancer.^8^ The differential host responses to *H pylori* colonization indicate that the pathogenicity of *H pylori* strains and other factors implicated in disease development.

It has been suggested that the cytotoxin‐associated gene A (CagA) gene with its products is linked to increased pathogenicity of *H pylori *strains. As one of the most virulent pathogenicity islands genes, CagA is able to perturb multiple host signaling pathways by acting as a hub or extrinsic scaffold protein, in turn potentiating malignant transformation.^9^ CagA can interact with multiple cell components and activate multiple signaling molecules downstream of receptor tyrosine kinase growth factors, therefore causing a set of complex cellular alterations, for example, enhanced proliferation and attenuated apoptosis, changes in epithelial cell morphology and polarity, and prevention of the assembly of apical junctions.^9^ A serologic response to CagA in *H pylori*‐infected patients was found to be strongly associated with peptic ulceration.^10^ Oncogenic potential of CagA has been proved in animal experiments.^11^, ^12^ People infected by cagA ‐positive H pylori strains have a higher risk of developing gastric carcinoma compared to those infected with cagA‐negative strins.^13^ CagA‐positive *H pylori* strains also participate in the inflammatory response, through the production of certain cytokines such as IL‐1β and IL‐8, and activation of NF‐κB.^14^ Thus, infection of CagA+ H* pylori* strains can alter both the local and systematic environments, which might consequently influence the local and distant microbiota in the host. Indeed, with a mouse model, the intestinal microbiota has proven to be altered by the presence of *H pylori* in the stomach.^15^

To date, the impact of *H pylori* infection on local and distant microbial populations in humans remains to be explored. In particular, it is essential to illuminate the role of non‐*H pylori* organisms in the development of gastrointestinal diseases, so as to improve diagnosis and treatment strategies. The oral microbiome helps host against invasion of opportunistic microorganisms. The oral microbiome also impacts the microbial communities that colonize the gastrointestinal tract,^16^ and its imbalances contribute to not only oral diseases but also risk of gastrointestinal disorders, adverse pregnancy outcomes, cardiovascular disease, diabetes, rheumatoid arthritis, and nervous systemic diseases.^17^ In this study, we sought to elucidate the relationships between CagA status of *H pylori *infection and the compositional, functional, and ecological changes in both gastric and oral microbiome communities.

## 2 MATERIALS AND METHODS

### 2.1 Cohort

Eighty Chinese participants diagnosed with chronic nonatrophic gastritis were enrolled in this study, who were diagnosed by gastroscopy referred to endoscopy center of The Traditional Chinese Medicine Hospital of Shijiazhuang, China, from June 2016 to February 2018. Indications for gastroscopy were recurrent abdominal pain, dyspepsia, and symptoms compatible with reflux esophagitis. These patients were not treated with antibiotics or proton‐pump inhibitors for at least 1 month before endoscopy. The presence of *H pylori* in antral gastric mucosa was determined by 16S rRNA gene sequencing, ^13^C‐urea breath test, and anti‐CagA immunoglobulin G (IgG) in serum. The patients were grouped accordingly as *H pylori*‐negative (n = 32), *H pylori*‐positive but CagA‐negative (n = 13), and *H pylori*‐positive and CagA‐positive (n = 35). The study protocol was approved by the ethics committee of The Traditional Chinese Medicine Hospital of Shijiazhuang. Demographic and diagnostic characteristics, and *H pylori*/CagA status of the enrolled patients are given in Table S1.

### 2.2 Sample collection, DNA isolation, and 16S rRNA gene sequencing

Sample collections were performed in the morning, and all the volunteers had fast (no food, fluids, or water) before the procedure. In order to overcome variations potentially caused by different gastric regions, all mucosal biopsy samples were collected from the antrum region of the stomach. Tongue coating samples were collected by scraping the tongue dorsum three times with sterilized cotton swabs. All samples were enclosed in sterile plastic tubes with RNAlater (Qiagen, German) immediately after collection and transported to HRK‐biotech laboratory with dry ice. Both gastric biopsy and tongue coating samples were stored at −80°C until tested.

Samples were centrifuged at 3420 X g for 10 minutes, and supernatant was removed. Homogenization was employed using 0.1 mm zirconium beads and bead beating for 5 minutes at 1500 *g* (Scientz‐48, High‐throughput Tissue Grinder, Scientz, China). Total DNA was isolated using QIAamp DNA Mini Kit (Qiagen, Germany), following the manufacturer's instructions. DNA was quantified using a Qubit dsDNA HS Assay Kit (Thermo Fisher Scientific, Waltham, MA, USA). PCR amplification and library preparation were accomplished using previously reported methods.^18^ In brief, amplification of the V3‐V4 hypervariable regions of the ribosomal 16S rRNA genes was performed using universal primers 341F (5′‐CCTACGGGAGGCAGCAG‐3′)/806R (5′‐GGACTACNNGGGTACTAAT‐3). Purified amplicon libraries were sequenced on the MiSeq platform (Illumina, Inc, San Diego, CA) using the 2 × 300 bp paired‐end protocol.

### 2.3 Data analysis

The forward and reverse reads were merged using VSEARCH^19^ with a minimum overlap set to 10 bp. Sequences were quality filtered at a maximum expected error of 1% and striped 5 bp (0‐5 bp heterogeneityspacers) at both sides. The USEARCH^20^ *cluster_otus* command was employed to filter chimeric sequences and cluster operational taxonomic units (OTUs) based on 97% nucleotide similarity. Taxonomic category was assigned to all OTUs using the Ribosomal Database Project (RDP) classifier.

Differential analysis was performed by SHAMAN (http://shaman.c3bi.pasteur.fr/) with a negative binomial generalized linear model (implemented in the DESeq2 R package), and the false discovery rate was adjusted by using Benjamini‐Hochberg procedure. The bacterial abundances were adjusted for potential confounding factors, including age, gender, BMI, and *H pylori* status. The microbiome *alpha* diversity was measured by the number of observed OTUs, the Shannon diversity index, the Simpson index, and the Inverse Simpson index. Overall differences in microbial community structure (ie, beta diversity) were evaluated through principal coordinate analysis (PCoA) and hierarchical clustering to Bray‐Curtis distance calculated at the OTU level.

Phylogenetic Investigation of Communities by Reconstruction of Unobserved States (PICRUSt) was used to predict metabolic functions of the microbial communities based on Greengenes (V13.5) 16S rRNA database and Kyoto Encyclopedia of Genes and Genomes (KEGG) orthologs.^21^ The functional differences between groups were determined with Linear Discriminant Analysis Effect Size (LEfSe). Organism level microbiome phenotypes were predicted and compared with BugBase.^22^ The proportions of six phenotypic categories, including Gram staining, oxygen tolerance, ability to form biofilms, mobile element content, pathogenicity, and oxidative stress tolerance, were compared among different groups of patients with gastritis.

### 2.4 Microbial association network analysis

SparCC algorithm^23^ was utilized to elucidate microbial interactions for each sample group. SparCC obtains the *P*‐value through permutation‐based approaches that iteratively correct spurious correlation coefficients, and limits false discovery rates. OTUs that occur in at least 50% of the samples within one group and with an average relative abundance of at least 1% were selected to infer the correlation network. Taxon‐taxon correlation coefficients were estimated as the average of 20 inference iterations and 200 shuffled matrices for the *P*‐value calculation. This set of iterative procedures was applied separately to each sample group of gastric mucosa and tongue coating ecologies. Correlation coefficients with magnitude ≥0.3 and *P*‐value <0.05 were selected for visualization in Cytoscape V.3.6.1.

## 3 RESULTS

### 3.1 Infection with CagA+ H pylori strains influences the gastric microbial community structure

It was shown that pyrosequencing method detected*H pylori *sequences in about 60% of samples that were *H pylori* negative by a combination of conventional testing (histology, rapid urease test, and culture),^24^ which emphasizes the use of NGS as a more sensitive method for determining *H pylori* infection. Following the proposed cutoff value of determining *H pylori* infection,^24^ we labeled samples with >1% *H pylori* relative abundance as *H pylori* positive (*H pylori*+). However, there is still a certain possibility to have false‐negative case due to uneven distribution of the organism through the gastric mucosa. To reduce the false‐negative rate, we also labeled samples with ≤1% *H pylori* relative abundance as *H pylori*+ if the patients were positive for both ^13^C‐urea breath test and serological CagA. The rest of samples were grouped as *H pylori* negative (*H pylori*−). We noticed that some low relative abundances of *H pylori* sequences were detected when breath test and/or serology were positive. This is likely due to the uneven distribution of *H pylori* in the stomach.

**Figure 1:**
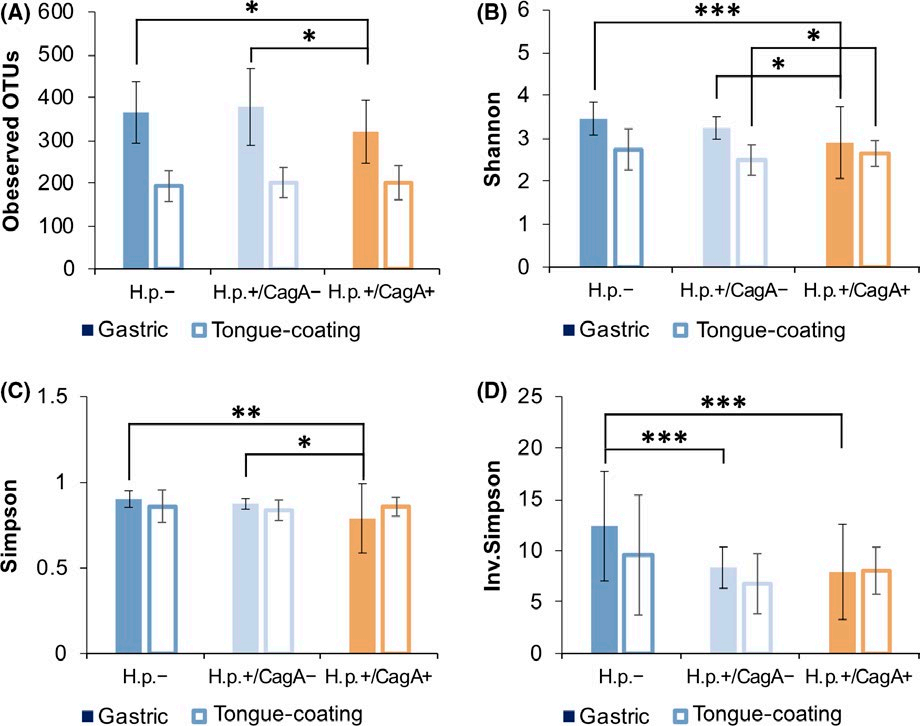
Microbial diversity and richness reduced in gastric biopsies of *H pylori*+/CagA+ samples. Bacterial community richness was defined by the observed number of OTUs (A), and alpha diversity was calculated using the Shannon index (B), Simpson index (C), and inverse‐Simpson index (D). Statistical significance was determined by paired student t‐test, *P < 0.05, **P < 0.01, and ***P < 0.001

**Figure 2:**
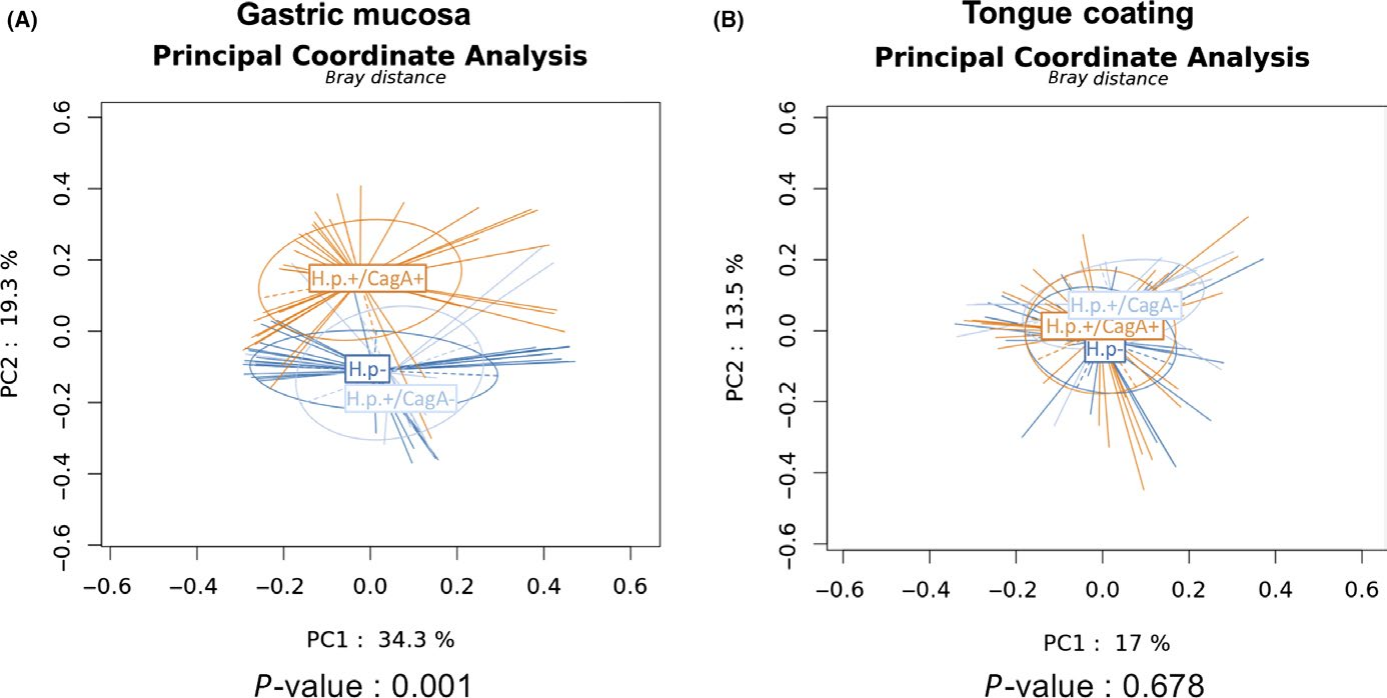
The influence of *H pylori* colonization on gastric and tongue coating microbial community structure. (A) The gastric microbiota profile differs in *H pylori*+/CagA+, *H pylori*+/CagA− and *H pylori*−. (B) The tongue coating microbiota is similar among the three patient groups. PCoA based on Bray‐Curtis distance is tested with PERMANOVA

The microbial diversity was measured and compared at the OTU level. A decreasing gastric bacterial *alpha* diversity from *H pylori*− through *H pylori*+/CagA− to *H pylori*+/CagA+ subjects was identified (Figure 1). The Shannon index, Simpson index, and Inverse Simpson index revealed a significantly lower gastric bacterial *alpha* diversity in the *H pylori*+/CagA+ compared to the other two groups. A similar pattern of decline in *alpha* diversity was also found in the tongue coating microbiota, but statistical significance was not obtained. Principal coordinates analysis (PCoA) of the gastric microbiota clearly separated the *H pylori*+/CagA+ group from the other two groups (Figure 2A), while the *H pylori*+/CagA− and *H pylori*− groups were similar to each other. The tongue coating microbiota composition was similar in all patient groups (Figure 2B). The results from PCoA are also consistent with the results of hierarchical clustering (Figure S1).

### 3.2 Relative microbial abundances in the human stomach are influenced by the presence of CagA+ H pylori strains

**Figure 3:**
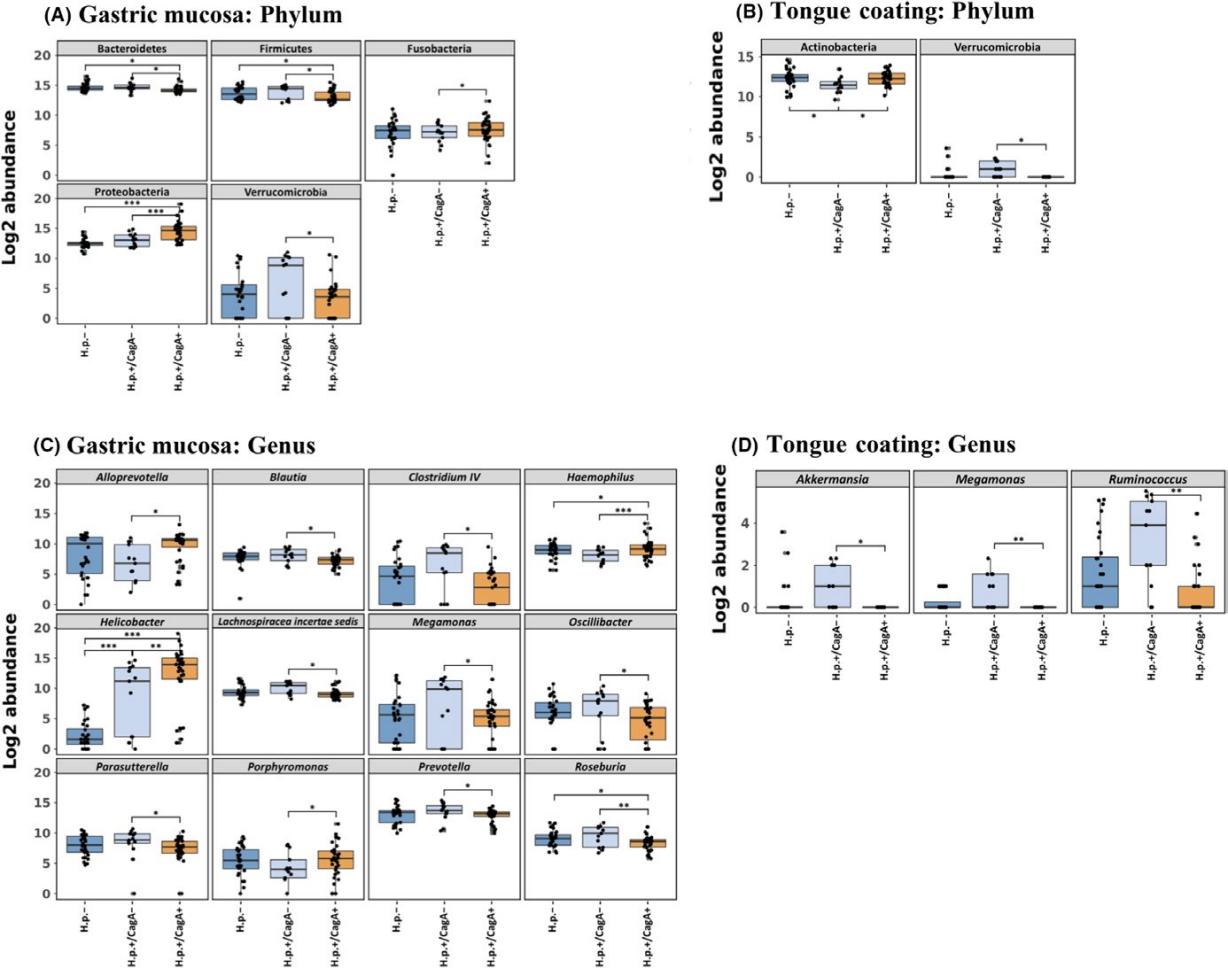
Alterations in the gastric and tongue coating microbiota composition were associated with *H pylori* infection. Bacterial phyla (A, C) and genera (B, D) demonstrated significantly different log2 abundances across different sample groups. Lower and upper limits of the boxes represent 25th and 75th percentiles, respectively. Whiskers represent 1.5* inter‐quartile range. *P* value ranges are: *P < 0.05, **P < 0.01, and ***P < 0.001

Bacterial profiles in the mucosa revealed that the gastric microbiota was mainly composed of Bacteroidetes (44.08%), Proteobacteria (27.49%), Firmicutes (23.3%), and Actinobacteria (3.78%) phyla (Table S2). The gastric bacterial flora of the *H pylori*+/CagA+ group was dominated by Proteobacteria (*H pylori* belongs to this phylum), while Bacteroidetes was the highest phylum in both *H pylori*+/CagA− and H.p.− groups. Bacteroidetes and Firmicutes were found to be significantly depleted in *H pylori*+/CagA+ compared with other two groups (Figure 3A). At the genus level, the *H pylori*+/CagA+ group had higher *Helicobacter* and *Haemophilus*, and lower *Roseburia* relative abundances compared to other groups (Figure 3C). Except for *Helicobacter*, no other genus was found with significantly different between *H pylori*− and *H pylori*+/CagA− groups. Additionally, significant differences in the relative abundances of two phyla (Fusobacteria and Verrucomicrobia) and nine genera (*Alloprevotella*,* Blautia*, *Clostridium*,* Lachnospiracea incertae sedis*,* Megamonas*,* Oscillibacter*,* Parasutterella*,* Porphyromonas*, *and Prevotella*) were found only between *H pylori*+/CagA+ and *H pylori*+/CagA− groups (Figure 3A,C).

As for the tongue coating microbiota, the most prevalent phyla were Bacteroidetes (30.74%), Proteobacteria (28.53%), Firmicutes (21.74%), Actinobacteria (11.12%), and Fusobacteria (6.29%) (Table S2). The proportion of Actinobacteria in the *H pylori*+/CagA− group was significantly lower than other groups (Figure 3B). The genera *Akkermansia*, *Meganomas*, and *Ruminococcus* were found to be more abundant in the *H pylori*+/CagA− compared with the *H pylori*+/CagA+ group (Figure 3D). Notably, *Meganomas* was found to be significantly more abundant in the *H pylori*+/CagA− compared with the *H pylori*+/CagA− group, for both gastric mucosa and tongue coating samples.

Taken together, these results indicate that *H pylori* infection, especially by the CagA+ strains, significantly altered the diversity and composition of gastric microbiome, while moderately affected the composition tongue coating microbiome.

### 3.3 Changes in the functional capacity of gastric microbiota is associated with *H pylori* infection and CagA gene expression

**Figure 4:**
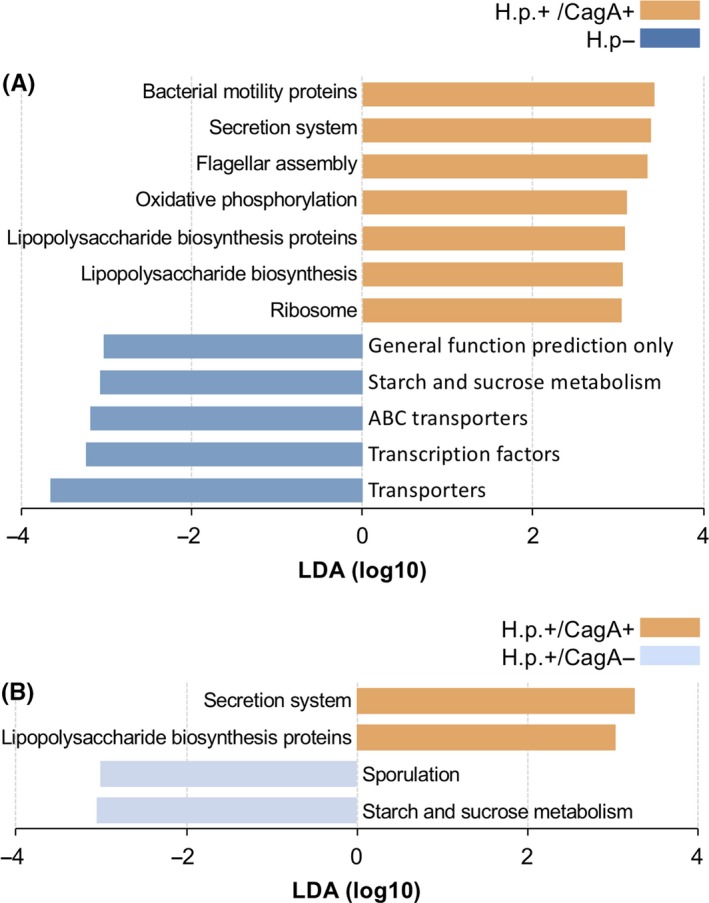
Changes in the function of gastric microbiota induced by *H pylori* infection. LEfSe identified the KEGG pathways with the significant differences in abundance (A) between *H pylori*+/CagA+ and *H pylori*−, and between (B) *H pylori*+/CagA+ and *H pylori*+/CagA− patients. Predetermined threshold on the logarithmic linear discriminant analysis (LDA) score for discriminative features was set at > 3.0; predetermined α‐value for the factorial Kruskal‐Wallis test was set at > 0.01

The functional capacities of the gastric mucosa and tongue coating microbiome were predicted based on 16S data using PICRUSt and BugBase. Statistically significant KEGG pathways for each group were determined using LEfSe. Compared to the H.p.− group, gastric microbiome of *H pylori*+/CagA+ patients was significantly enriched for pathways involved in bacterial motility proteins, secretion system, flagellar assembly, oxidative phosphorylation, lipopolysaccharide (LPS) biosynthesis proteins, LPS biosynthesis, and ribosome (Figure 4). In addition, the secretion system and LPS biosynthesis proteins were also upregulated in the *H pylori*+/CagA− group. No significant difference in functions was detected between the *H pylori*− and *H pylori*+/CagA− groups.

**Figure 5:**
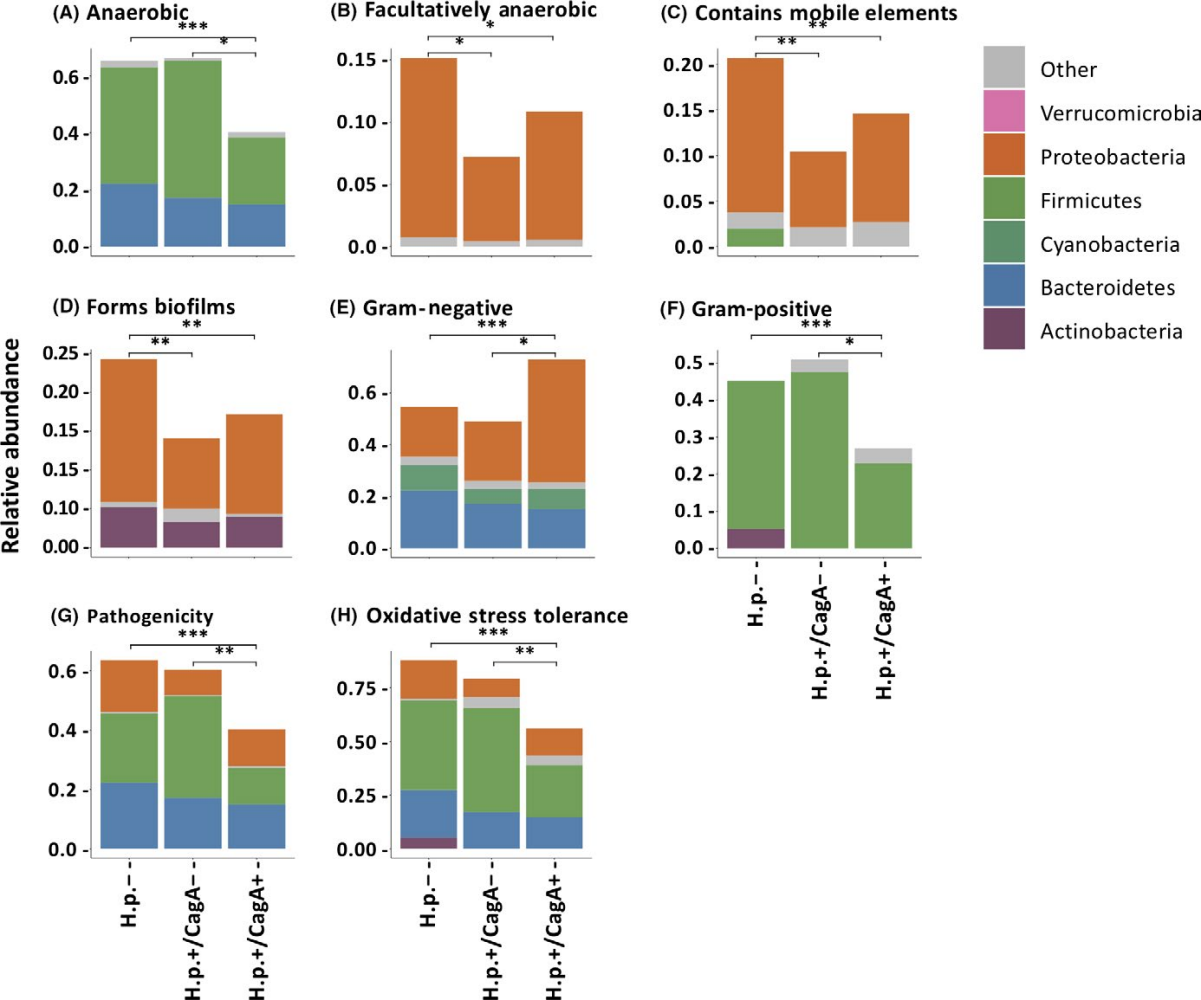
BugBase predicted microbial community phenotypes and the corresponding bacterial contributions. (A–B) Oxygen utilization. (C) Mobile genetic element content. (D) Biofilm formation. (E–F) Gram bacterial classification. (G) Pathogenic risk. (H) Oxidative stress tolerance. Statistical significance was determined by Mann‐Whitney U test. P value ranges are: *P < 0.05, **P < 0.01, and ***P < 0.001

At the organism level, gene functions related to facultative anaerobiosis, mobile element containing, and biofilm forming were depleted in the *H pylori*+ samples, and mainly due to some reduced taxa that belong to the genus Proteobacteria (Figure 5B‐D). Gram‐negative species were significantly enriched in *H pylori*+/CagA+ samples, likely owing to the increased abundance of *H pylori* (Figure 5E). In addition, significant reductions of anaerobic, Gram‐positive, potential pathogenic, and oxidative stress tolerant microorganisms were predicted for the *H pylori*+/CagA+ sample group, which were majorly attributed to the decreased abundance of Firmicutes (Figure 5A,F‐H).

Significant alteration in the functions of tongue coating microbiome was not obtained. Thus, *H pylori* infection significantly influenced the gene functions of gastric microbiota, and the presence of CagA gene made a further contribution to this alteration.

### 3.4 Alterations of gastric and tongue coating microbial interactions are associated with *H pylori* infection and CagA gene expression

The human body contains many diverse microorganisms competing and cooperating with one another, thus acting as an ecosystem supporting health or promoting disease. Most of microbial interactions are niche‐specific^25^; therefore, the disease‐specific microenvironment may also shape microbial interaction networks. We next inferred taxonomic correlations among the three sample groups, using the SparCC algorithm. OTUs that occur in ≥50% of the samples within one group and with a relative abundance ≥1% were selected to infer the correlation network.

**Figure 6:**
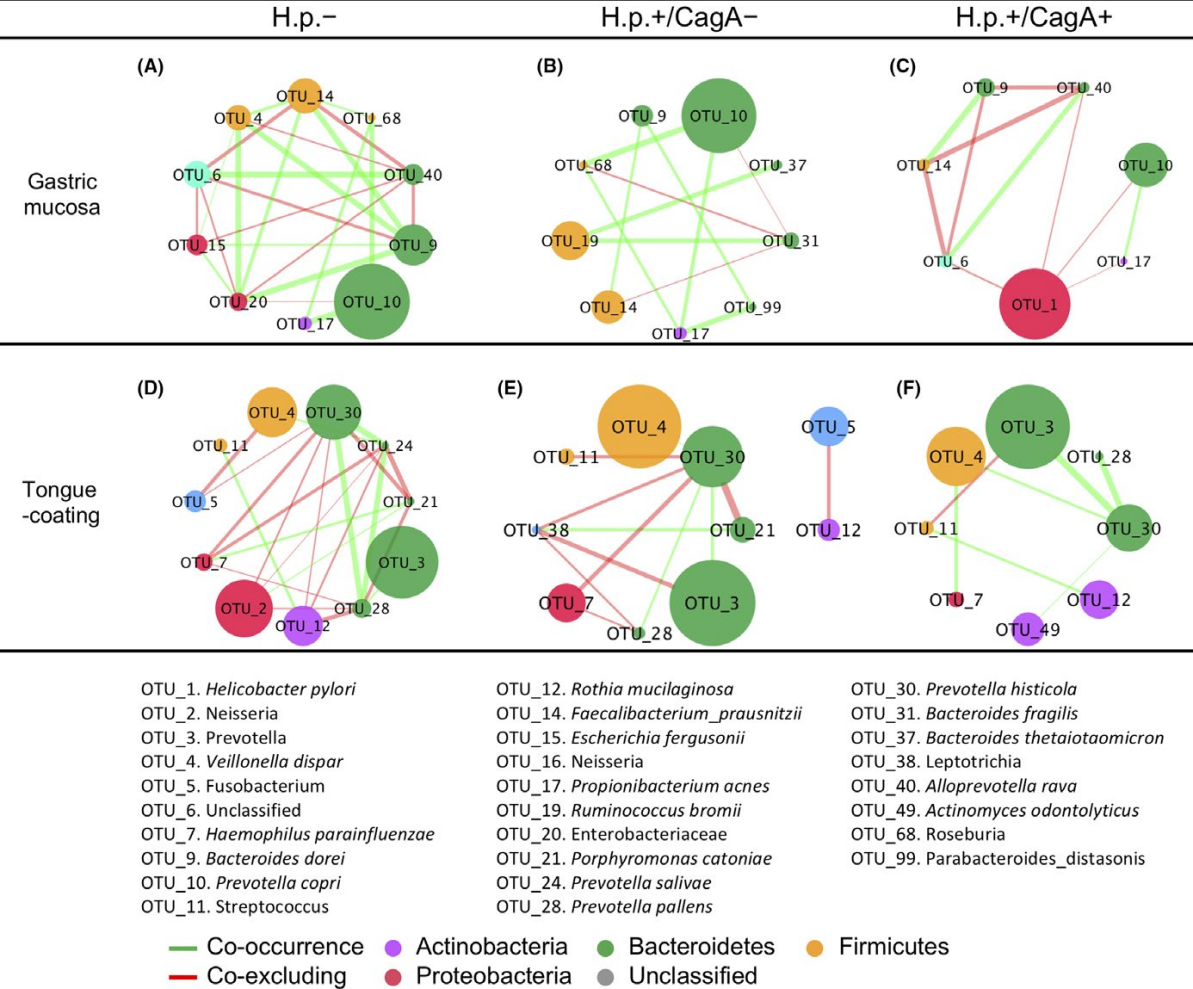
H pylori infection altered the gastric and tongue coating microbial interaction networks. Correlation networks of gastric (A‐C) and tongue coating microbiota (D‐E) in *H pylori*−, *H pylori*+/CagA−, and *H pylori*+/CagA+ were inferred by using SparCC. Visualization was applied to a subset of correlations with strengths greater than 0.3. Node size represents mean relative abundance of taxon in each sample group; metacommunity markers are denoted by node numbers accordingly. Node colors indicate the members of the same bacterial phylum

Among taxa colonizing the gastric mucosa, the number of interactions was highest in the *H pylori*− group (n = 24), which was significantly reduced in the *H pylori*+ subjects (*H pylori*+/CagA−: n = 11; *H pylori*+/CagA+: n = 11) (Figure 6A‐C). Co‐occurrence between OTU_9_*Bacteroides dorei* and OTU_14_*Faecalibacterium prausnitzii*, and between OTU_10_*Prevotella copri* and OTU_17_*Propionibacterium *were found to be ubiquitous in all the groups. The interactions between *H pylori *and other taxa were exclusively co‐excluding in the *H pylori*+/CagA+ samples (Figure 6C). Some interactions were shared between H.p.− and H.p.+/CagA− sample groups, such as co‐occurrence between OTU_10_*Prevotella copri* and OTU_68_*Roseburia*, and between OTU_17_*Propionibacterium* and OTU_68_*Roseburia*, which were depleted in the H.p.+/CagA+ group. The interaction pattern among OTU_6_Unclassified, OTU_9_*Bacteroides dorei*, OTU_14_*Faecalibacterium prausnitzi*, and OTU_40_*Alloprevotella rava *was consistent in H.p.− and H.p.+/CagA+ groups (Figure 6A,C). Co‐occurrence among OTU_19_*Ruminococcus bromii*, OTU_31_*Bacteroides fragilis*, and OTU_37_*Bacteroides thetaiotaomicron* were uniquely observed in the *H pylori*+/CagA− group (Figure 6B).

The number of bacterial interactions within the tongue coating microbiota also decreased from *H pylori*− (n = 22) to H.p.+ samples (H.p.+/CagA−: n = 12; H.p.+/CagA+: n = 7) (Figure 6D‐F). OTU_30_ *Prevotella_histicola* exhibited co‐occurrence relationships with OTU_4_*Veillonella dispar* and OTU_28*_* *Prevotella pallens*, which were found to be ubiquitous in all sample groups. The network of *H pylori*+/CagA+ group was dominated by cooperation relationships, and only one negative correlation (between OTU_3_*Prevotella* and OTU_11_*Streptococcus*) was identified (Figure 6F). Interactions of OTU_7_Roseburia with OTU_28*_* *Prevotella pallens* and OTU_30_ *Prevotella_histicola *that found in the *H pylori*− and *H pylori*+/CagA− groups were depleted in the *H pylori*+/CagA+ group.

Taken together, infection with *H pylori*, especially the CagA+ strains, was associated with a reduced complexity of bacterial interactions in both gastric and tongue coating microbial communities. As a result, the oral microbiome structure is more likely to be disturbed such as by invasion of alien microorganisms, since the community response hinged on the complex interspecific interactions.

## 4 DISCUSSIONS

Former studies on mice gastric microbiome indicated that *H pylori* infection significantly affects the population structure of the gastric and intestinal microbiota, alters gastric immune and inflammatory responses, and causes distant effects via altered hormones and immunity.^15^ Because physiology and immunity were substantially influenced following the infection of *H pylori*, we asked whether the induced host response affects microbiome composition distally in the mouth. In this exploratory study, we characterized both the gastric and tongue coating microbial patterns in patients with gastritis and investigated their relations to *H pylori* infection with and without presence of the CagA gene. In concordance with previous descriptions, we found Actinobacteria, Bacteroidetes, Firmicutes, and Proteobacteria were the most prevalent phyla in the gastric specimens.^1^, ^2^ Multiple taxa were found to be significantly different between *H pylori*− and *H pylori*+/CagA+ (but not H.p.+/CagA−) subjects, together with a reduced microbial diversity, indicating the presence of CagA gene was strongly linked to alterations in the gastric microbiota. It was previously shown that gastric microbiome of *H pylori*+/CagA+ patients was not significantly different compared to *H pylori*+/CagA−,^4^ which was not in concordance with our results. It could be attributed to insufficient sample size in both studies (10 CagA− and 10 CagA+ samples in 4; 13 CagA− and 35 CagA+ samples in our study).

The metabolites of gut microbiota play crucial roles in determining the biochemical profile of the diet, and thus modulating host metabolism. However, the metabolism of gastric microbiota (especially non‐*H pylori* species) and their interplay with host metabolism in health and disease remain poorly understood. We found that the functions involved in LPS biosynthesis were upregulated within patients infected by CagA+ strains. LPS (also termed endotoxin) is the major surface molecule of most Gram‐negative bacteria that play a key role in host‐pathogen interactions with the innate immune system. Indeed, the proportion of Gram‐negative taxa was significantly higher in the stomach of *H pylori*+/CagA+ patients. In the gastric mucosa, LPS is capable of suppressing the elimination of *H pylori* by interfering the activity of innate and adaptive immune cells, diminishing the inflammatory response, and affecting the adaptive T lymphocyte response, thus facilitating the development of chronic gastritis.^26^ Additionally, the exposure of LPS has been found in close associations with several diseases such as endotoxemia, autoimmune and allergic disease,^27^ obesity,^28^ and nervous systemic diseases (including autism^29^ and Alzheimer's disease^30^). It has been hypothesized that most of LPS is derived from the gut microbiota, and enters blood circulation due to an increased intestinal barrier permeability. However, it is also possible for LPS to across the gastric mucosa into blood circulation, especially for patients with gastric lesions. Thus, together with the vascular permeability in gastric mucosal, LPS overexpression in the stomach should be further investigated.

As an open system, the oral cavity contains a variety of microbial habitats including teeth, tongue, gingival sulcus, cheek, and palates which contribute to a vast ecological complexity. The diversity and composition of tongue coating have been found more stable comparing to teeth and supragingival plaque which were more susceptible to oral hygiene habits.^31^ In addition, most of the highly abundant taxa found in saliva were derived from the tongue. Therefore, we collected tongue coating samples to access the oral microbiota in this study. Although *H pylori* infection had a subtle influence on the microbial diversity and structure of tongue coating microbiota, the complexity of bacterial interaction network was greatly reduced. Our data indicated that the total number of interactions were significantly declined in the oral microbiota of the *H pylori*+ group, especially for the CagA+ patients. Additionally, oral microbiota of CagA+ patients was dominated by co‐occurrence relationships, further indicating a low network complexity since cooperation is destabilizing for the community.^32^ Thus, the oral microbiota of CagA+ patients may be more tolerant to the invasion of alien species.

In conclusion, our study identified the influences of *H pylori* infection on gastric and tongue coating microbial populations. Infection with CagA+ *H pylori* strains reduced the diversity of gastric microbiota and altered its composition and functions. The upregulation of LPS biosynthesis likely resulted from Gram‐negative bacteria enrichment in the stomach of CagA+ patients may increase the chance of developing various diseases that associated with LPS exposure. CagA+ *H pylori* infection was also associated with an unstable tongue coating bacterial community, which suggests a weak resistance to invasion by microorganisms with pathogenic potential.

## DISCLOSURES OF INTERESTS

The authors declare no competing financial interests.

## AUTHORS’ CONTRIBUTIONS

GX analyzed data and drafted the manuscript. GJ, XY, WH, and LY collected human samples. GJ and YD performed DNA isolation and sequencing. ZY designed the study and revised the paper.
