# Supplementary figures and images for: Helicobacter pylori infection alters gastric and tongue coating microbial communities
Source: Helicobacter. 2019 Feb 7;24(2):e12567. doi: 10.1111/hel.12567 (PMC6593728; doi:10.1111/hel.12567)

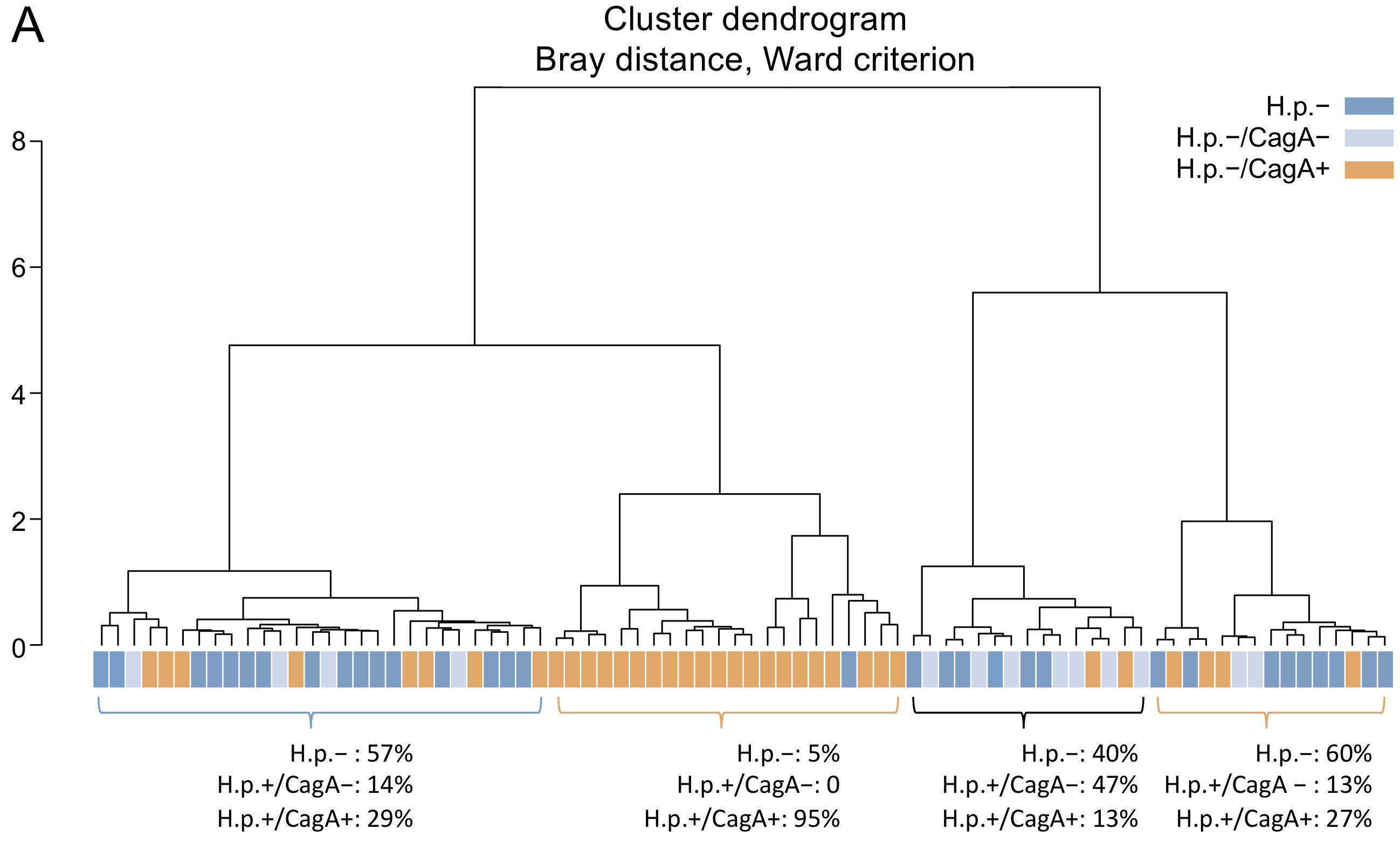

Supplement: Supplementary file 1 [file HEL-24-na-s001.png]

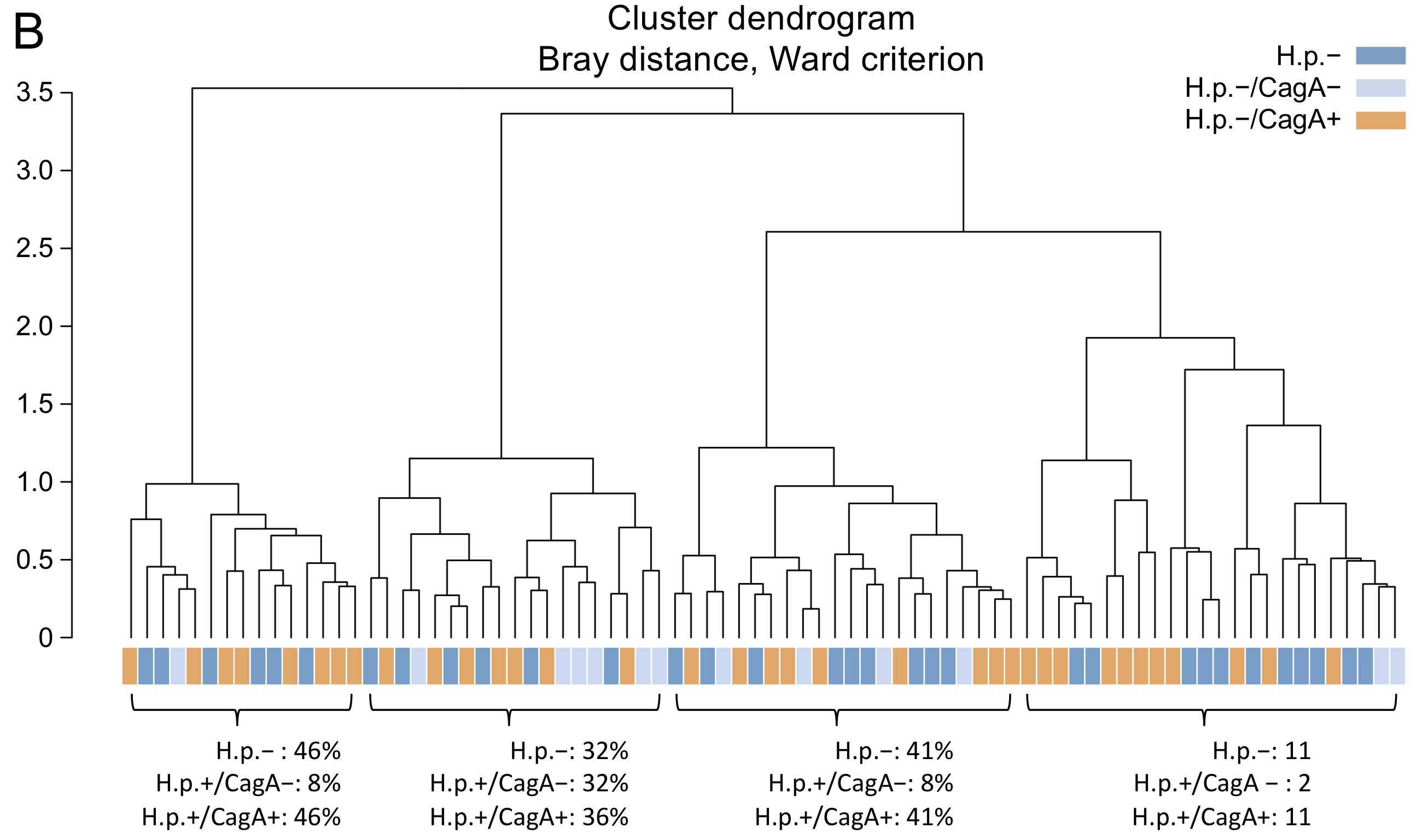

Supplement: Supplementary file 2 [file HEL-24-na-s002.png]
